# PMS: A Panoptic Motif Search Tool

**DOI:** 10.1371/journal.pone.0080660

**Published:** 2013-12-04

**Authors:** Hieu Dinh, Sanguthevar Rajasekaran

**Affiliations:** Computer Science and Engineering Department, University of Connecticut, Storrs, Connecticut, United States of America; CSIR-Institute of Microbial Technology, India

## Abstract

**Background:**

Identification of DNA/Protein motifs is a crucial problem for biologists. Computational techniques could be of great help in this identification. In this direction, many computational models for motifs have been proposed in the literature.

**Methods:**

One such important model is the 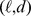 motif model. In this paper we describe a motif search web tool that predominantly employs this motif model. This web tool exploits the state-of-the art algorithms for solving the 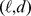 motif search problem.

**Results:**

The online tool has been helping scientists identify many unknown motifs. Many of our predictions have been successfully verified as well. We hope that this paper will expose this crucial tool to many more scientists.

**Availability and requirements:**

Project name: PMS - Panoptic Motif Search Tool. Project home page: http://pms.engr.uconn.edu or http://motifsearch.com. Licence: PMS tools will be readily available to any scientist wishing to use it for non-commercial purposes, without restrictions. The online tool is freely available without login.

## Introduction

Motif search is an important problem in biology. Computational techniques could greatly help in solving this problem. A number of computational motif search tools can be found in the literature. See e.g., PRATT [1], MEME [2], DILIMOT [3], SLiMDisc [4], SLiMFinder [5] and FIRE-pro [6].

Each of the above tools is based on a specific model of motif search. An important model for motifs is the 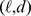-motif search model. A simple version of this model can be stated as follows. We are given 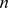 input sequences 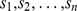 each of length 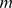. Input are also two integers 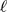 and 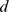. The problem is to find a motif 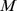 that is present in the 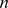 input sequences. It is known that 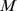 is of length 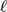 and that it occurs in each of the 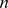 input sequences within a Hamming distance of 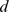.

This model has been shown to yield better sensitivities than that of other models when tested on known biological data (see e.g., [7]). The problem of 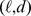-motif search is intractable [8]. There are numerous algorithms that have been proposed for solving the 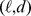-motif search problem. Examples are RISO and RISOTTO [9]. But RISO and RISOTTO are down-loadable programs and there are no corresponding web systems. In this paper we describe a web system for motif search that uses the 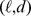-motif model. Our web system has the following features: 1) We employ several state of the art algorithms for 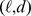-motif search. We can identify longer motifs than RISO and RISOTTO. RISO can only identify motifs of length up to 14. PMS can identify motifs of length up to 23; 2) Both DNA and protein motifs are supported; 3) We support quorum motif search. In this case the motif(s) need not occur in all the input sequences. Quorum motif search is significantly more difficult than the regular version [10]; 4) Dyads motifs are also found. In particular, the dyad motif under concern could consist of two segments separated by a gap; 5) We employ a scoring mechanism for the putative motifs found; and 6) The user interface for PMS is very friendly; 7) In PMS, user emails are optional.

To the best of our knowledge, there is no other comprehensive motif search system, based on the 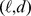-motif model, comparable to ours.

## Results

### The PMS Webserver

The PMS server is freely available at http://pms.engr.uconn.edu or at http://motifsearch.com. The website is open to any user. Login is not required. However, any user with a login account will have the benefit of viewing and retrieving his or her submission(s) history. Also, a submission associated with a registered user will be kept in the system forever unless the user deletes it. Any submission from a user without a login account will be stored in the system for one month. It will be automatically removed after one month.

The purpose of the motif search tool is to help biologists identify novel motifs that may be present in input DNA and/or Protein sequences. Simple and user-friendly input forms will allow users to submit queries easily and quickly. Informative output and visualizations will permit users to analyze the results carefully. These features of the website are described in more detail in the following sections.

### Input Sequences and Parameters

The input data can be either DNA or protein sequences. The length of each sequence is required to be between 15 and 1000. The number of input sequences is required to be between 5 and 500. The input sequences should be organized in the well-known text-based format - FASTA.

**Figure 1:**
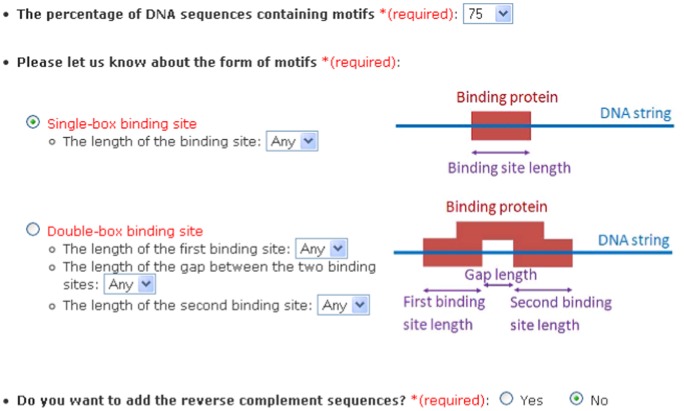
Parameters for DNA sequences. The set of required parameters for DNA sequences. The first parameter is “*quorum percent*” which is the minimum percentage of the input sequences containing motifs. The second parameter allows users to choose the structure of motifs.

For each input dataset, a set of parameters will be chosen by the user. These parameters are shown in Figure 1. The first parameter is called “*quorum percent*” which is the minimum percentage of the input sequences that contain motifs. Quorum percent is set to 75% by default.

The second parameter allows users to choose the structure of motifs. Currently, the tool considers two structures, namely, monads and dyads. A monad is a contiguous string and a dyad consists of two segments separated by a gap. A monad is assumed by default. For monads, the users will choose the motif length. By default, the motif length is chosen to be “Any” which means that the tool will search for motifs of lengths between 10 and 25. If information about the motif length is known, we recommend that it be used to reduce the processing time. For dyads, users should choose the length of the first segment or box, the length of the second box and the length of the gap between the two boxes. If the lengths are chosen to be “Any”, processing will proceed similar to that for monads.

The third parameter is for DNA sequences that allows users to have the option of considering the reverse complement sequences. If the input DNA sequences have the same orientation, the third parameter should be chosen to be “No”. Otherwise, we recommend that it be chosen to be “Yes”.

### Submitting Jobs

**Figure 2:**
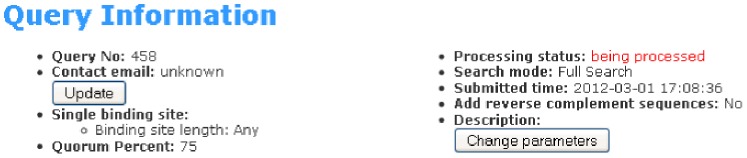
Query information. Information about submission. Users can click on the “Update” button or the “Change parameters” button to update the contact email or change parameters.

After entering the sequences and relevant parameters, the user clicks on the “Submit” button on the submission form. If the data entered are valid, the submission will enter the processing queue. Once the processing is over, a results page will be displayed. Information about the submission will appear on top of the results page as shown in Figure 2. Users can update contact email or change the parameters by clicking on either the “Update” button or the “Change parameters” button, respectively.

**Figure 3:**
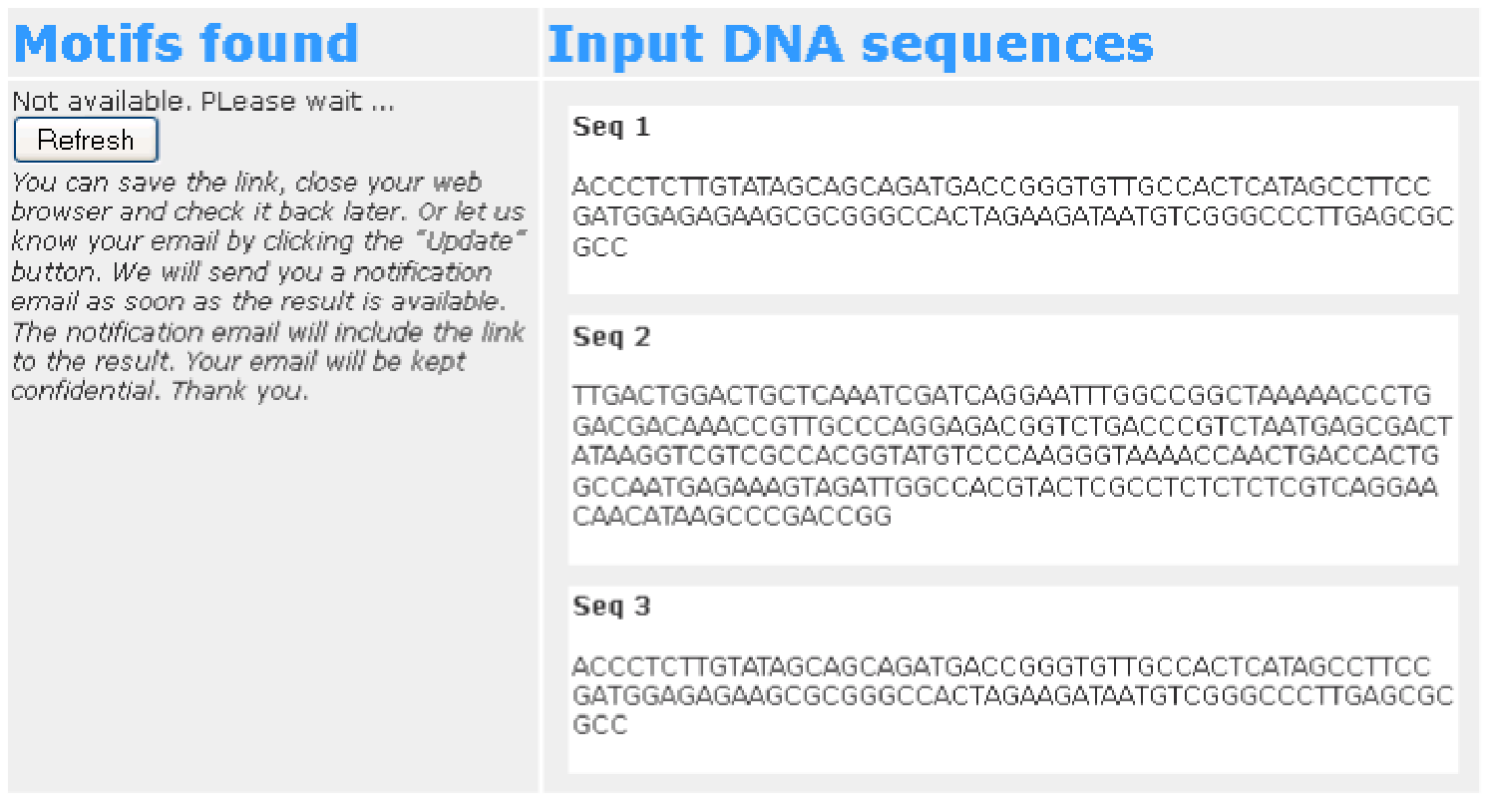
Result not available. An example of the results page when the submission has not been processed yet. Users can click on the “Refresh” button to update the processing status.

After submission, the submission status could be one of these: in processing queue, being processed, and processed. If the submission has not been processed yet, the bottom of the results page will appear as shown in Figure 3. Users can click on the “Refresh” button to update the processing status. Users can either wait for their submission to be processed or bookmark the results page and return to it later. If the contact email is provided, the system will send a notification email when the submission is processed. The notification email will include the URL for the results page.

The processing time of any submission varies from a few minutes to a few hours, depending on the data, the parameters, and the workload of the server. If the user feels that the tool is taking too much time to process, we recommend that (s)he provide his/her contact email. Providing emails has a number of benefits. The first benefit is that the user will receive email notifications when query processing is complete. The second benefit is that their submissions will be stored in the system as long as they want. The third and perhaps the most important benefit is that they can retrieve their submission histories (as discussed in the next section).

### Output

**Figure 4:**
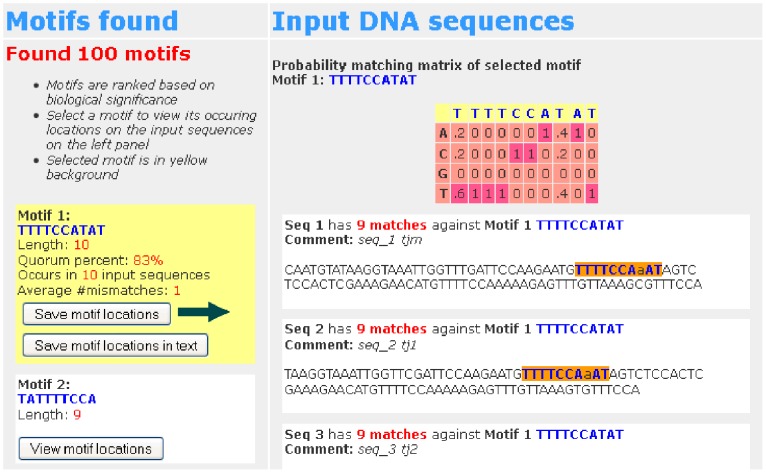
Result available - DNA sequences. An example of the results page when the submission is processed. The locations of the second motif are marked on the DNA input sequences.

Once the submission is processed, the bottom of the results page will appear as shown in Figure 4. Identified candidate motif(s) will appear on the left and the input sequences will appear on the right. If no motifs are found, we recommend to reduce the value of the quorum percent.

The candidate motif(s) found are ranked according to their scores. The score of a candidate motif is the logarithm of the probability that the motif occurs by random chance. The smaller the score, the more biologically significant the motif is. For more details on the scoring scheme, the readers are referred to [10]. For each candidate motif, users can click on the “View motif locations” button corresponding to the motif in order to view its locations, i.e., its instances, in the input sequences. The locations of the motif instances will be highlighted in the input sequences as shown in Figure 4. The probability weight matrix of the motif is directly calculated through its motif instances and will appear above the input sequences. The probability of a DNA character at each column in the probability weight matrix is its frequency when its motif instances are aligned. When a motif is chosen, users can click on the “Save motif locations in text” button to save its locations in a text file.

**Figure 5:**
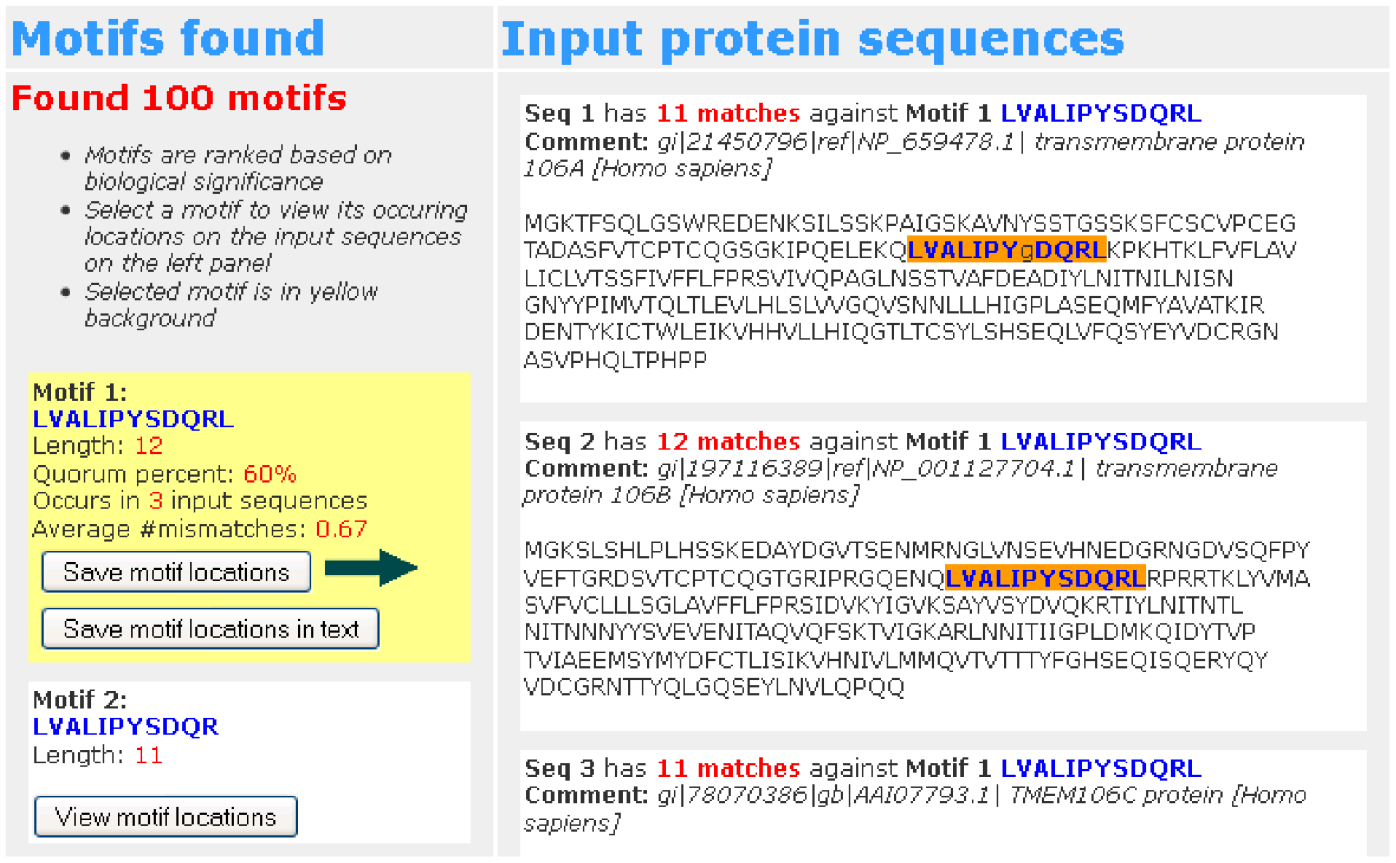
Result available - Protein sequences. An example of the results page when the submission is processed. The locations of the second motif are marked on the protein input sequences.

For input protein sequences, the results are shown in Figure 5 which is similar to that of DNA sequences except that the probability weight matrix is not shown because it would be large for protein sequences.

### Submissions History

The website allows users to easily manage their submission(s) history. To start the submissions history feature, click on the link “Submission history” on the left menu of the website. To view submissions history, enter the contact email and password on the submissions history form. If the password has not been set by a user yet, (s)he can go to the reset password form and enter the contact email. An email will be sent to the contact email including a URL that allows the user to reset the password.

**Figure 6:**
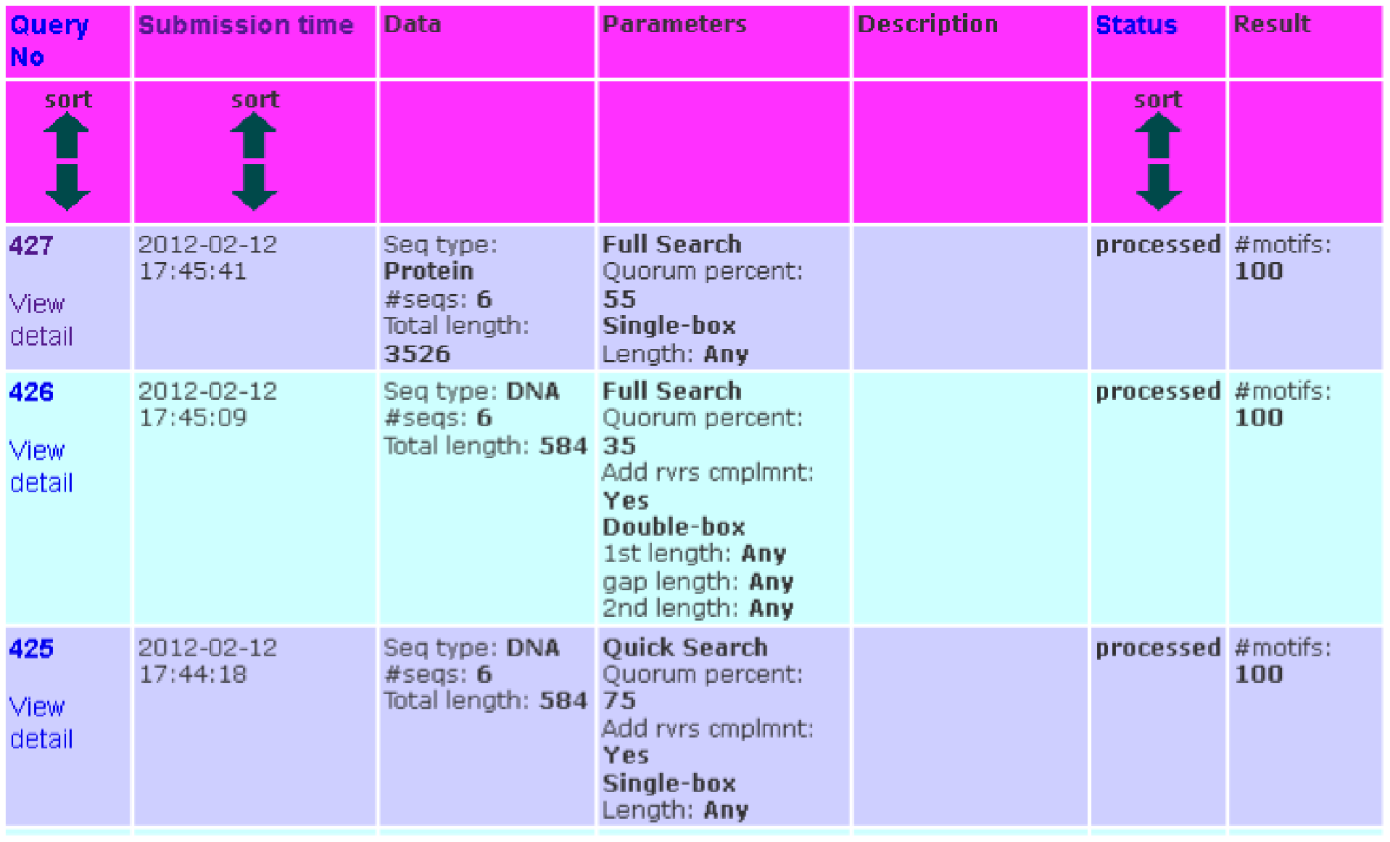
Submission history. An example of the submissions history. Users can sort their submissions based on query ID, submission time, or processing status. Users can view a particular submission in detail by clicking on the link “View detail” of the according submission.

The list of submissions will be shown as in Figure 6. Users can sort their submissions based on query ID, submission time, or processing status. If the users want to view a particular submission, they can click on the link “View detail” of the corresponding submission.

### Feedback

The website supports an extensive feedback section. Users can easily submit feedbacks, comments, and questions using the feedback form. Feedbacks and comments will help us improve the website. To access the feedback form, click on the link “Feedback” on the left menu of the website.

## Discussion

In this paper we have described a new web tool for motif search called PMS. This tool is based on the 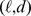-motif search model. This is a comprehensive web tool offering many crucial features and we are not aware of any other computational motif search tool comparable to ours. In future we plan to support additional features. For example, we will identify candidate motifs with more than two segments (separated by gaps). Another important feature will be to score the candidate motifs based on experimental data publicly available. User feedbacks will also be taken into account in enhancing the features of our web tool PMS. We also plan to incorporate other motif models in future. In addition we plan to work on finding longer motifs.

## Materials and Methods

Our online motif search tool is built on state-of-the-art algorithms for the most well-known motif model - 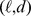-motif search or the Planted Motif Search (PMS). The PMS model has been shown to be very effective in identifying motifs (see e.g., [7]). The PMS Problem is defined as follows.

**Definition 0.1** ***PMS Problem*** *: given 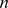 sequences and integer parameters 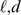 and 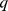, find all strings 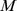 of length 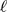 such that 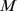 appears in at least 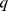 out of the 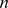 given sequences within 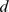 mutations. Each such string 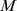 is a putative motif. Any 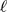-mer (i.e., a substring of length 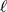) 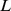 in any input string such that the Hamming distance between 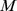 and 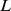 is at most 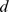 is known as an instance of the motif* 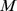.

### The PMS Algorithms

In our web tool, we have used a combination of the current best PMS algorithms proposed in [10], [11], and [12].

We now summarize some of the techniques used in these algorithms.

Let 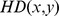 stand for the Hamming distance between two strings 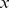 and 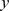 of the same length. Let 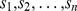 be the given set of input sequences each of length 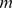. For simplicity, consider the version where 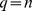. The PMS0 algorithm works as follows [13]: Consider 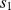. Let 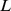 be an 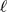-mer of 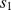. Define the 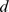-neighborhood 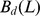 of 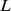 to be the collection of all the 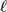-mers 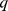 such that 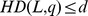. If 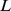 is an instance of an 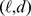-motif 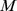, then, clearly 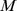 will be in 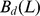. However, we do not know which 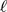-mers of 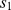 are instances of the motif we are looking for. Thus, PMS0 constructs 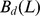 for every 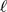-mer 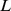 in 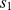. It then performs a union 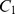 of all of these 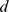-neighborhoods. 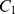 contains all the 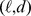-motifs. For each 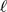-mer 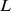 in 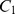, the algorithm checks if 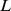 is an 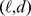-motif or not in an obvious manner. Note that for a given 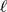-mer 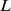, we check if it is an 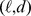-motif or not in 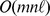 time. A variation of this algorithm is called PMS1 and is described below [13]:

### Algorithm PMS1

1. Compute 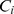 for each input sequence 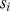, 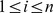. Here 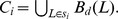 In other words, 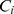 is nothing but the union of 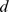-neighborhoods of all the 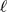-mers in 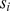, 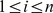. The notation 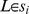 indicates that the 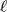-mer 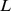 is a substring in 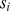.
2. The 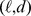-motifs are now computed as 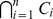.

Algorithm PMS5 can be thought of as an extension of PMS0 [11]. If 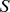 is a collection of strings, let 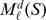 denote the 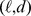-motifs present in 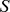. If the input sequences are 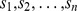, let 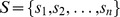 and let 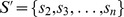. The idea of PMS5 is to compute the 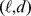-motifs of 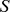 as 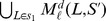.

In order to compute 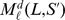 for any 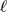-mer 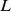, the algorithm uses a subroutine to compute the common 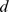-neighborhood of three 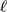-mers. Specifically, let 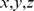 be any three 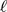-mers. We use 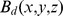 to denote the common 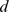-neighborhood of 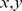, and 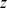. In other words, 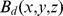 is nothing but the set of all 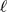-mers that are at a distance of no more than 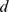 from each of the three 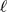-mers 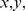 and 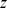.

To compute 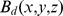, PMS5 represents 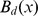 as a tree 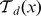. Each node in this tree is an 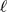-mer in 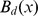. The root of 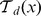 is the 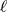-mer 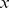. The depth of 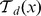 is 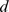. 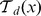 is traversed in a depth-first manner. Let 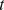 be any node in this tree. During the traversal, 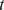 will be output if 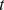 is in 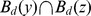. While visiting any node 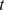, we check if there is a descendent 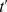 of 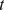 such that 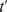 is in 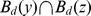. The subtree rooted at 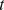 will be pruned if there is no such descendent. The problem of checking if 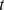 has any descendent that is in 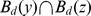 is formulated as an integer linear program (ILP) on ten variables. This ILP is solved in 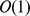 time.

Any algorithm for solving the PMS problem when 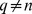 is typically named with a prefix of ‘q’. One of the first algorithms to address this version of the PMS problem was qPMSPrune [12]. Algorithm qPMSPrune is based on the following observation: If 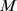 is any 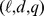-motif of the input strings 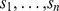, then there exists an 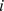 (with 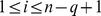) and an 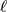-mer 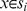 such that 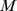 is in 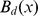 and 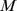 is an 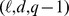-motif of the input strings excluding 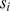. The algorithm runs through every possible value of 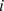, 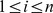. For a given value of 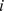, it considers every 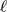-mer 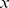 of 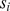. Specifically, it constructs 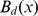 and identifies elements of 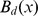 that are 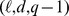 motifs (with respect to input strings other than 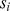). 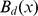 is represented as a tree with 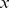 as the root. This tree is traversed in a depth first manner and some pruning conditions are used to prune subtrees that do not have any motifs.

Algorithm qPMS7 of [10] extends the observation of qPMSPrune as follows: If 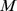 is any 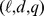-motif of the input strings 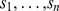, then there exist 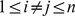 and 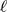-mer 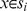 and 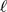-mer 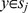 such that 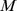 is in 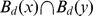 and 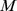 is an 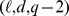-motif of the input strings excluding 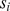 and 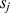. qPMS7 considers every possible pair 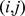, 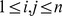 and 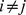. For a given pair 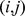, every possible pair of 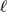-mers 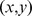 is considered (where 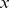 is from 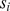 and 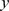 is from 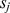). For a given 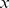 and 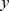, the algorithm finds all the elements of 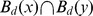 that are 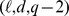 motifs (with respect to input strings other than 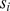 and 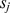). 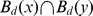 is explored by traversing an acyclic graph, denoted as 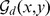. 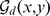 is traversed in a depth first manner. Here again effective pruning conditions are used to prune subgraphs of 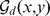.

For more details about the PMS algorithms, the readers are referred to the respective papers.

### An Experimental Validation of PMS Algorithms

Planted motif search is just one computational model for motifs. An important question is how efficient is this model in identifying motifs from real biological data. In fact the same question is relevant for any (computational or other) motif model. In [14], Tompa, et al. have evaluated the performance of 13 different motif finding programs: AlignACE, ANN-Spec, Consensus, GLAM, The Improbizer, MEME, MITRA, MotifSampler, Oligo/dyad-analysis, QuickScore, SeSiMCMC, Weeder and YMF. These programs were evaluated on several biological datasets (for which the motifs were known via experimental techniques) based on many different performance measures. Two of the performance measures employed were sensitivity and specificity. Sensitivity represents the fraction of sites that were correctly predicted and specificity represents the fraction of non-sites that were correct.

In [7], Sharma, et al. have evaluated the performance of PMS algorithms. In particular, they have employed the same 56 datasets that were used by Tompa, et al. [14]. As a result, Sharma, et al. have compared the PMS algorithms with the thirteen programs evaluated in [14]. Several versions of the PMS algorithms have been tested. One of these versions, namely, PMS SumMinD yields an average sensitivity of 28.8% and a specificity of 91.63% on all the 56 datasets. In comparison, the best of the 13 algorithms tested by Tompa, et al. [14], ANN-Spec, has an average sensitivity of 8.7% and a specificity of 98.22%.

### Our Motif Search Framework

In addition to the PMS algorithms, we deploy a motif search framework that uses the PMS algorithms as underlying routines. The motif search framework basically works as follows. The user inputs a set of sequences that contain motifs of interest. The framework runs a PMS algorithm (qPMS7 as of now) with different triples of the parameters 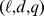 and collects all of the output motifs. These motifs are called candidate motifs. Then, it uses a score function that ranks the candidate motifs. The score function measures the significance of a candidate motif based on the probability that it occurs by random chance. Finally, the tool outputs the top 100 motifs with the highest scores. The score of a candidate motif will be high if the probability that it occurs by random chance is low.

Since the run time of PMS algorithms is exponentially dependent on the parameter 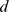, i.e. maximum number of mutations allowed, we let the user indirectly set the parameter through the computational preferences, “Quick Search” or “Full Search”. If the “Quick Search” option is chosen, then the parameter 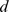 is set to a ‘low’ value (3, specifically). Conversely if the “Full Search” option is chosen, then the parameter 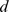 is set to a higher value (7, specifically).

### Identifying Motif Instances in the Input Sequences

Once a motif is found, its instances in the input sequences will be located as follows. For each input sequence, the location of the motif instance in the input sequence is the place where the motif matches the most. The motif location can be done easily by scanning through the entire input sequence.

### Techniques to Identify Dyad Motifs

Eskin and Pevzner have presented an algorithm for finding dyads motifs [15]. This algorithm works as follows. Let the input sequences be 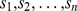 and let the length of each sequence be 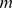. A dyad is characterized with the parameters 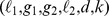. Here 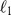 is the length of the first segment, 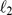 is the length of the second segment, the length of the gap between the two segments can be in the range 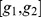, and the dyad occurs in at least 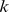 out of the 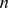 sequences with a Hamming distance of at most 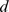. For each input sequence 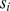, the algorithm generates all the relevant 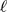-mers (where 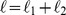). Any such 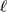-mer will be such that its prefix of length 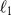 will be an 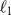-mer in some input sequence 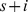, its suffix of length 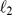 will be an 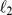-mer in the same sequence 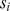, the prefix occurs to the left of the suffix, and the length of the gap between the prefix and the suffix is in the range 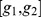. Note that there are 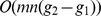 such 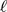-mers. Let 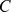 be this collection of 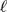-mers. After having generated these 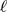-mers, they use the mismatch tree data structure to identify the 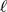-mers that correspond to valid dyads. In particular, any 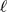-mer will be output as a dyad if there is a 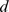-neighbor of this 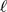-mer that occurs in at least 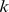 of the input sequences.

We speed up the above algorithm exploiting the PMS1 algorithm. The improvement works as follows. We generate the 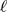-mers for each sequence as in the algorithm of [15]. There are 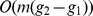 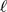-mers for each sequence. Let 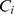 be the collection of 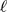-mers from sequence 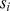, for 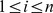. For each 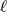-mer of 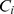 generate its 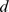-neighborhood (i.e., 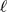-mers that are within a Hamming distance of 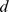 from the 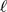-mer), for 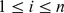. Let 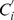 be the collection of 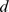-neighbors of all the 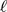-mers of 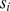, for 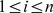. We can output 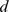-neighbors that are in at least 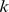 of these collections. One way of finding such 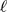-mers will be with the help of hashing. Another way is to make use of integer sorting. For example, we can sort each 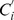 (for 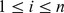), merge these sorted lists, and go through the merged list to count the number of sequences each such 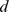-neighbor occurs in.

### Availability and Requirements

Project name: PMS - Panoptic Motif Search Tool. Project home page: http://pms.engr.uconn.edu or http://motifsearch.com. Licence: PMS tools will be readily available to any scientist wishing to use it for non-commercial purposes, without restrictions. The online tool is freely available without login.
